# Pre-conditioned mesenchymal stem cells ameliorate renal ischemic injury in rats by augmented survival and engraftment

**DOI:** 10.1186/1479-5876-10-243

**Published:** 2012-12-05

**Authors:** Muhammad Shareef Masoud, Sanam Saiqa Anwar, Muhammad Zeeshan Afzal, Azra Mehmood, Shaheen N Khan, Sheikh Riazuddin

**Affiliations:** 1 National Centre of Excellence in Molecular Biology, University of the Punjab, Lahore, Pakistan; 2 Current Affiliation: Department of Bioinformatics and Biotechnology, Government College University, Faisalabad, 38000, Pakistan; 3 College of Medicine- Pathology and Laboratory Medicine, University of Cicinnati Ohio, Albert Sabin way, Cicinnati, USA; 4 Allama Iqbal Medical College/Jinnah Hospital Complex, University of Health Sciences, Lahore, Pakistan; 5 The University of Lahore, 1Km Raiwind Road Lahore, Lahore, Pakistan

**Keywords:** MSCs, SNAP, Pre-conditioning, Renal ischemia, Cytoprotective factors

## Abstract

**Background:**

Ischemia is the major cause of acute kidney injury (AKI), associated with high mortality and morbidity. Mesenchymal stem cells (MSCs) have multilineage differentiation potential and can be a potent therapeutic option for the cure of AKI.

**Methods:**

MSCs were cultured in four groups SNAP (S-nitroso N-acetyl penicillamine), SNAP + Methylene Blue (MB), MB and a control for *in vitro* analysis. Cultured MSCs were pre-conditioned with either SNAP (100 μM) or MB (1 μM) or both for 6 hours. Renal ischemia was induced in four groups (as in *in vitro* study) of rats by clamping the left renal padicle for 45 minutes and then different pre-conditioned stem cells were transplanted.

**Results:**

We report that pre-conditioning of MSCs with SNAP enhances their proliferation, survival and engraftment in ischemic kidney. Rat MSCs pre-conditioned with SNAP decreased cell apoptosis and increased proliferation and cytoprotective genes’ expression *in vitro*. Our *in vivo* data showed enhanced survival and engraftment, proliferation, reduction in fibrosis, significant improvement in renal function and higher expression of pro-survival and pro-angiogenic factors in ischemic renal tissue in SNAP pre-conditioned group of animals. Cytoprotective effects of SNAP pre-conditioning were abrogated by MB, an inhibitor of nitric oxide synthase (NOS) and guanylate cyclase.

**Conclusion:**

The results of these studies demonstrate that SNAP pre-conditioning might be useful to enhance therapeutic potential of MSCs in attenuating renal ischemia reperfusion injury.

## Background

The mammalian kidney has multifarious functions, including maintenance of blood pressure, regulation of pH and other metabolic processes, such as elimination of nitrogenous wastes and synthesis and release of endocrine factors. Loss of regional or total blood flow to the kidney often happens in shock, sepsis and during renal transplantation, can lead to acute kidney injury (AKI). Inflammatory process and primary ischemic injury in vascular cells results in prolonged localized ischemia that substantially increases severity of injury in affected areas. During ischemia, the endothelium produces free radicals and secretes chemo-attractants which upon reperfusion sequester and activate neutrophils and intensification of the injury [1]. AKI associated mortality is estimated as 50% in hospitalized patients and 70-80% in patients under intensive care [2,3]. Currently, the therapeutic choices are confined to supportive measures and preventive strategies. None of these have shown a decrease in mortality rate caused by AKI. Kidney transplant is an alternate option but scarcity of donor; high cost and immune rejection limits its utility. It is therefore imperative to find other therapeutic options that could regenerate the tubular epithelium, restore kidney function and reverse the effects of AKI.

MSCs are multi-potent cell population residing in bone marrow. These are capable of differentiating into cell types other than their tissue of origin. In culture, these cells are characterized by their unique ability to adhere to the plastic surface and exhibit fibroblast like morphology [4]. Moreover MSCs secrete an array of growth factors as vascular endothelial growth factor (VEGF), insulin like growth factor-1 (IGF-1), hepatocyte growth factor (HGF) and anti-apoptotic cytokines [5]. The therapeutic effects of MSCs after transplantation are dependent on their survival in the recipient tissue. Taking into account that prolonged cell survival may augment the usefulness of stem cell therapy. It has been reported that pre-conditioning of different tissues by a prior stimulus provides protection against injury [6]. The most extensively studied pre-conditioning stimulus is brief sub-injurious ischemia, which induces protection against subsequent ischemic injury [7]. Similar protective effects can also be triggered by growth factors, cytokines or with some pharmacological agents [5,8].

Nitric oxide (NO) is a free radical generated as a result of oxidation of L-arginine catalyzed by all three isoforms (neuronal, inducible and endothelial) of nitric oxide synthase (NOS) and performs different patho-physiological functions in different cellular environments [9-13]. It protects macrophages, hepatocytes, cardiomyocytes against different injuries by modifying expression of different genes [14]. Various donors of NO have been implicated as cyto-protective and tissue protective factors *in vivo*[1,15].

This study was designed to evaluate the effects of pre-conditioning of bone marrow derived MSCs with a pharmacological agent S-nitroso N acetyl penicillamine (SNAP, a NO donor) on the regenerative potential of MSCs against oxidative and ischemic stress in kidney. We report that SNAP pre-conditioning of MSCs improved their potential against oxidative stress, enhanced their proliferation and reduced apoptosis *in vitro*. Further it augmented the homing of MSCs in ischemic renal parenchyma, induced different cytoprotective and paracrine factors and improved renal function in “rat model of renal ischemic insult”.

## Materials and methods

### Animal care

The investigation conforms to the *Guide for the Care and Use of Laboratory Animals* published by the US National Institutes of Health (NIH Publication No. 85–23, revised 1985). All animals were treated according to the procedures approved by the Institutional Review Board (IRB) at the National Center of Excellence in Molecular Biology, Lahore, Pakistan.

### *In vitro* study

#### Isolation of MSCs from bone marrow

MSCs were isolated on the basis of their preferential attachment to plastic surface of culture flask as previously described [16]. Bone marrow MSCs were isolated by flushing femurs and tibiae of male Sprague–Dawley rats. The suspension was then centrifuged at 1200 rpm for 10 min at room temperature. The cells were plated in tissue culture flask containing Iscove’s Modified Delbeco’s Medium (IMDM, MP Biomedicals, USA) supplemented with 20% fetal bovine serum (Sigma-Aldrich, Germany), 100ug/ml streptomycin and 100U/ml penicillin and placed in incubator having 95% humidity and 5% CO_2_. The medium was changed after three days of plating and washed with phosphate buffered saline (PBS). The medium was then changed after every three days. When confluency is reached, the cells were sub-cultured in a ratio of 1:3 till they reached 70%-80% confluency. The cells of passages 2–3 were used in the study.

#### Pre-conditioning of stem cells

The cells were plated in tissue culture plates in equal number (2x10^5^) and divided in four groups. Group 1: control; cells incubated in normal serum free medium, group 2: cells treated with 100uM S-nitroso N acetylpenicillamine (SNAP) a NO donor, group 3: cells treated with both 100uM SNAP and 1uM methylene blue (an inhibitor of NOS and gyanalate cyclase) and group 4: the cells treated with 1uM methylene blue alone (group 4).

#### Measurement of lactate dehydrogenase, cell viability and apoptosis assay

Cell viability was assessed after exposing above mentioned groups of cells to 200μl H_2_O_2_ for 1 hour (hr). The medium was removed and trypan blue solution (Sigma-Aldrich, Germany) was added to the culture. The cells were then analyzed under phase contrast microscope [17]. The number of trypan blue positive cells was then counted per field for each treatment. LDH release was analyzed using cell supernatants by a commercially available kit (Sigma-Aldrich, Germany). Apoptosis was assessed by annexin V binding assay (Abcam, UK). The slides were then analyzed under the microscope (BX61 Olympus). The cells were counted manually and percentage of annexin V positive cells was calculated in each treatment group.

#### Measurement of release of nitric oxide

NO release in the culture medium was assessed from the amount of nitrite in the medium which is a stable reaction product of NO with molecular oxygen. 100μl of culture supernatant was treated with equal volume of Griess reagent (0.5% sulfanilamide, 0.05% N-1-naphthyl ethylenediamine dihydrochloride in 2.5% H_3_PO_4_) in 96 well plate and placed on shaker for 10 min at room temperature. The resulting color product was spectrophotometrically quantified at 538nm.

#### Measurement of gene activity

The effects of pre-conditioning on gene expression were assessed by performing quantitative real-time polymerase chain reaction using ABI Real-Time system 7500. All four groups of cells were exposed to H_2_O_2_ for 1 hr and then medium was aspirated and RNA from all four groups of cells was extracted using TRIZOL (Invitrogen, USA) according to the manufacturer’s instructions. cDNA was synthesized using 1μg of total RNA from each group by Revert Aid H Minus first strand cDNA synthesis kit (Fermentas).

The expression of genes shown in Table 1 was analyzed through real-time quantitative PCR, stromal derived factor-1 (SDF1), IGF1, AKT-1, VEGF, Bcl-2, PCNA and β-actin was used as an internal control using Maxima syber green qPCR mix (Fermentas) according to manufacturer’s protocol on 7500 thermal cycler (Applied Biosystem, USA). The relative gene expression normalized with β-actin and analysis was done by using SDS 3.1 software provided by Applied Biosystems, USA.

**Table 1:** Primer sequences of various genes

**Genes**	**Primer sequence**	
BCL-2	Forward,	5′-CGACTTTGCAGAGATGTCCA-3′
	Reverse,	5′-ATGCCGGTTCAGGTACTCAG-3′
VEGF	Forward,	5′-GCCCTGAGTCAAGAGGACAG-3′
	Reverse,	5′-GAGGAGGAGGAGCCATTACC-3′
IGF1	Forward,	5′-GCTGAAGCCGTTCATTTAGC-3′
	Reverse,	5′-CCACCCAGTTGCTATTGCTT-3′
PCNA	Forward,	5′- GACCTCGCTCCCCTTACAGT -3′
	Reverse,	5′- TCCAGCACCTTCTTCAGGAT -3′
SDF-1α	Forward,	5′- AGCCAGTCAGCCTGAGCTAC-3′
	Reverse,	5′- GGCACAGTTTGGAGTGTTGA-3′
HIF-1α	Forward,	5′-CTAGGGATGCAGCACGATCT–3
	Reverse,	5′-AGATGGGAGCTCACGTTGTG–3′
AKT 1	Forward,	5′-CCTCAAGAACGATGGCACCT-3′
	Reverse,	5′-CAGGCAGCGGATGATAAAGG-3′
β-Actin	Forward,	5′- GCTGTGTTGTCCCTGTATGC-3′
	Reverse,	5′- GAGCGCGTAACCCTCATAGA-3′

### *In vivo* study

#### Renal ischemic insult model

All animal procedures were according to instructions of institutional committee for animal care. Renal ischemic model was developed in male Sprague Dawley rats (200-300g) by blocking the left renal pedicle. Briefly, the rats were anesthetized with pentobarbital (40mg/kg body weight). A left lateral abdominal incision was made exposing kidney. The renal pedicle was occluded with micro vascular clamp maintaining body temperature at 37°C. The clamp was then removed after 45 minutes and kidney was reperfused. The peritoneal cavity and skin were closed with 4/0 suture. The animals were allowed to recover and returned to their cages.

#### Labeling of MSCs and transplantation in ischemic kidney

To track the transplanted cells and to evaluate the yield of transplantation *in vivo*, the cell suspension was labeled with CM-Dil (invitrogen, USA), a lipophilic dye that binds to cell membrane irreversibly.

Male rats were divided into five groups (n=6) after induction of renal ischemia to receive intra-renal parenchymal injection of either normal MSCs without pre-conditioning (MCs group), or SNAP pre-conditioned MSCs (SNAP group): methylene blue pre-conditioned MSCs (MB group) and SNAP plus methylene blue pre-conditioned MSCs (SNAP+MB group) and serum free medium injected control group (control). After 45 min ischemia to the kidney, 1.5 × 10^6^ labeled cells in serum free medium, were transplanted into renal parenchyma at three different sites just after reflow to each group described above while the control animals received only serum free medium. The incision was closed with suture and animals were revived and transferred to animal facility.

#### Collection of urine and blood

The rats were kept in metabolic cages and 24hrs urine was collected and measured. Blood was drawn through tail vein and serum was isolated for each rat.

#### Immunoflourescence staining

After 3 weeks of transplantation, animals were sacrificed and kidneys were harvested and processed. Frozen tissue sections (5 μm thickness) were air dried and washed with PBS for 5 min. Sections were incubated with specific primary antibody of actin (abcam, UK) with specified dilution after non-specific blocking with 2% donkey serum and then FITC (flourocinisothiocynate) conjuagated donkey anti mouse secondary antibody. The sections were counter stained with DAPI and mounted (prolong gold anti-fade, Invitrogen, USA). Fluorescence images were taken by Olympus BX 61 microscope with DP 70 camera.

#### Immunohistochemical analysis of ischemic renal tissue

Paraffin sections of kidney from all experimental groups were analyzed for the detection of different factors through application of enzyme labeled polymer (Vector ImmPRESS kit, Vector Laboratories, USA) according to manufacturer’s instructions. Briefly, the sections were subjected to citrate based antigen retrieval followed by endogenous peroxidase and avidin-biotin blocking using a Streptavidin-Biotin blocking reagent (Abcam, UK). Following serum blocking, sections were incubated either for one hour at room temperature or overnight at 4°C in humid conditions with the following primary antibodies of specified dilutions: a Rabbit polyclonal anti iNOS antibody, mouse monoclonal to VEGF, rabbit polyclonal to Ki-67, rabbit polyclonal anti eNOS antibody (Abcam, UK), rabbit polyclonal to IGF1 and rabbit polyclonal Bcl-2 (Santa Cruz, USA) followed by HRP conjugated secondary antibodies. The chromogen substrate 3, 3’-Diaminobenzidine Tetra hydrochloride (DAB) was incubated until the characteristic color was developed followed by hematoxylin counterstaining.

#### Measurement of collagen

Paraffin sections were stained with Sirius red to analyze fibrosis in the tissue. The slides were analyzed under BX61 microscope. Total collagen content was measured as the percent of area with the help of Image J (NIH) software.

#### Measurement of creatinine clearance and Blood Urea Nitrogen (BUN)

Creatinine was measured in 24 hr urine and serum of the rats using Crea Plus kit (Roche, Germany) according to the manufacturer’s procedure. Briefly the urine samples were diluted 1:50 and undiluted serum samples were used. The samples were analyzed @ 546 nm test wave length and @ 700 nm reference wave length and creatinine values were calculated as follow:

CreatinineClearance=creatinineofurine×24hrurinevolume×1440÷creatinineofserum.

The blood urea nitrogen level in serum was measured using a urea estimation kit (Diasys, Germany) according to the manufacturer’s procedure. Briefly serum was isolated from blood of each rat. The reagent was mixed with samples and analyzed at 340 nm wavelength by spectrophotometer. BUN was calculated as follow:

BUN=ODofsample×concentrationofstandard×0.47÷ODofstandard

#### Statistical analysis

All experiments were performed in triplicates and repeated at least three times. Statistical significance was analyzed using one way ANOVA followed by Bonferroni testing through Graphpad Prism 5 software. A *p*-value ≤ 0.05 was considered statistically significant.

## Results

### *In vitro* study

#### Cytoprotective effects of pre-conditioning on MSCs

**Figure 1:**
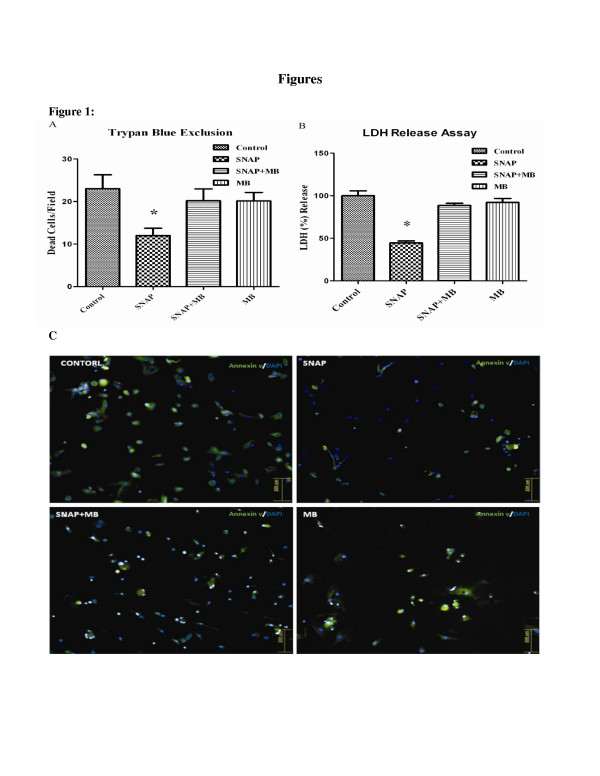
**Effect of SNAP pre-conditioning on viability of MSCs against hypoxic injury.A**: H_2_O_2_ induced cell death was decreased in SNAP pre-condition compared to the control and other groups. **B**: LDH release was decreased in SNAP pre-conditioned group compared to other treatment groups **C**: Annexin V staining of different pre-conditioned groups (magnification 200X). All values were expressed as mean ± SEM.

MSCs showed significant loss of viability when exposed to H_2_O_2_ (200 μM) for 1 hr. After SNAP pre-conditioning, cytotoxic effects of H_2_O_2_ were markedly decreased compared to non pre-conditioned control. This effect was diminished in SNAP/MB and MB alone groups (Figure 1A). Likewise SNAP pre-conditioned MSCs were distinctly protected against H_2_O_2_ as shown by decreased release of LDH as compared to control group (Figure 1B). Apoptosis in MSCs after pre-conditioning was assessed by annexin V staining. The percentage of annexin V positive cells decreased from 27% in control group to 9% in SNAP pre-conditioned group (Figure 1C). This decrease in cell death was diminished with methylene blue treatment (22% and 23% in SNAP/MB and MB groups respectively).

#### Release of nitric oxide in medium

The amount of nitrite produced as a result of reaction of NO with molecular oxygen was estimated. Griess reagent was added to the medium and OD at 538 nm wavelength was taken. Level of nitrite was significantly high (2.59 ± 0.3 μM) in the medium of SNAP group compared to the control (0.27 ± 0.2 μM).

#### Enhanced Expression of Cytoprotective, Proangiogenic and proliferating genes after SNAP pre-conditioning

**Figure 2:**
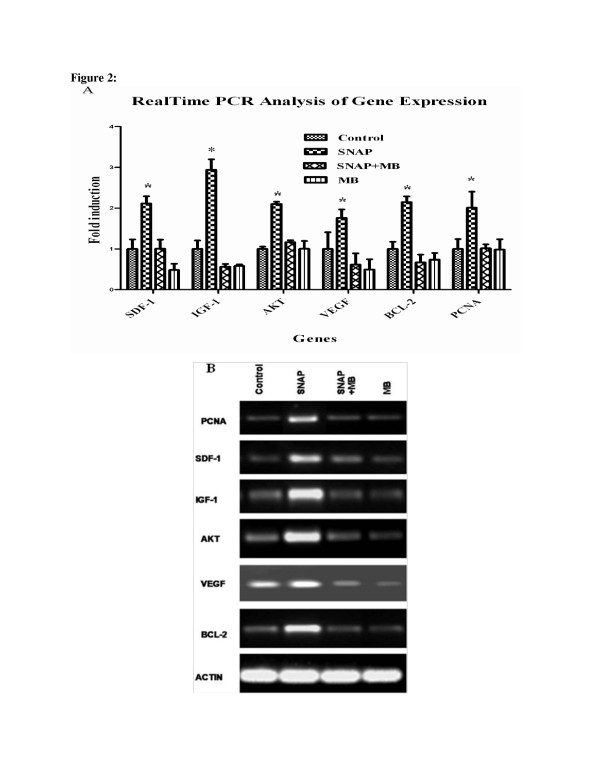
**Gene Expression Analysis of pre-conditioned MSCs.** Real time PCR was performed to relatively quantify the expressions of cytoprotective and proangiogenic genes in different groups after pre-conditioning. The expression of cytoprotective and proangiogenic genes was more pronounced in SNAP pre-conditioned group compared to other treatment groups. **A**: graphical presentation of fold induction in expression of various genes and **B**: agarose gel electorphoresis pictures of different genes.

The quantitative analysis of cyto-protective and pro-angiogenic growth factor genes by real time PCR revealed many folds increase (2.11 ± 0.178, 2.94 ± 0.026, 2.01 ± 0.052 and 1.76 ± 0.21) in the expression of SDF1, IGF1, Akt and VEGF respectively in SNAP pre-conditioned group (Figure 2A and B) compared to normal MSCs, SNAP+MB and MB gourps. Similarly, B cell lymphoma 2 (Bcl-2) a cytoprotective gene expressed under the influence of Akt [18] was up-regulated after SNAP pre-conditioning (Figure 2A and B).

Effect of SNAP pre-conditioning on proliferation of MSCs was assessed by analyzing the expression of proliferating cell nuclear antigen (PCNA) by real-time RT-PCR. Significant increase in the expression of PCNA (Figure 2A and B) was observed in SNAP treated group (2.012 ± 0.242) when judged against control (1 ± 0.241).

### *In vivo* studies

#### Pre-conditioning enhanced homing of transplanted MSCs in ischemic renal tissue

**Figure 3:**
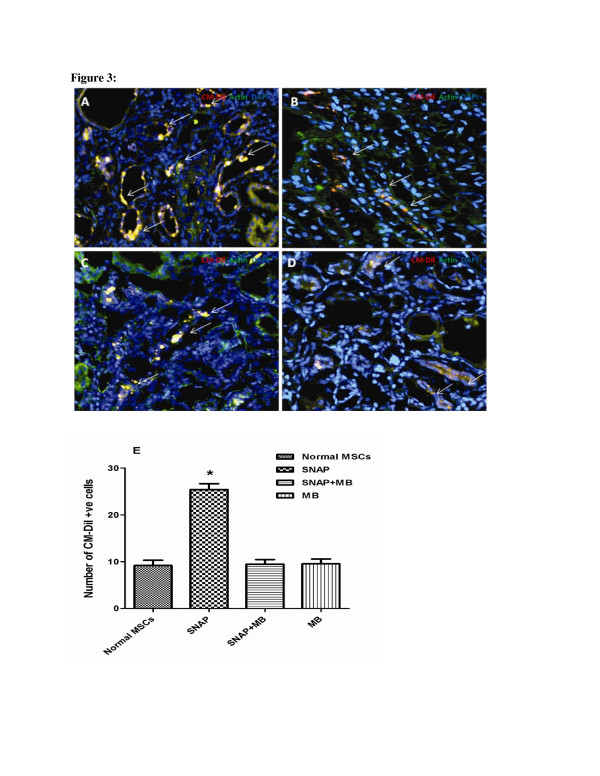
**Effect of pre-conditioning on homing of MSCs in ischemic renal tissue.** Homing of pre-conditioned MSCs in ischemic kidney tissue was assessed by tracking the CM-Dil positive cells under fluorescence microscope. The tissues were stained with actin (green), CM-Dil in red and nuclei were stained with DAPI (blue). Extensive homing of the CM-Dil positive MSCs was observed in SNAP pre-conditioned group (**A**) while a limited number of CM-Dil positive cells were observed in non pre-conditioned MSCs group (**B**). Whereas fewer MSCs were observed in SNAP/methylne blue and methylene blue only treated groups relative to the SNAP pre-conditioned group (**C** &**D**), magnification = 200x. **E** is graphical presentation of cell homing.

A significant increase in the homing of CM-Dil labeled MSCs was detected at the site of injured tubular cells in SNAP pre-conditioned group compared to control (22.0 ± 1.0 vs. 7.0 ± 1.0; Figure 3). The homing of MSCs reduced to 10.0 ± 1.0 and 8.0 ± 1.0 CM-Dil positive cells per field in SNAP/MB and MB groups (Figure 3). All values were expressed as mean ±SEM.

#### Effect of Pre-conditioning on renal function and fibrosis

Administration of SNAP pre-conditioned MSCs ameliorated the kidney function as assessed by sustained improvement in creatinine clearance (Table 2) and decreased blood urea nitrogen levels over a period of three weeks compared to other treatment groups.

**Table 2:** Renal function (creatinine clearance and blood urea nitrogen) analysis of rats

**Treatments/Parameters**	**Creatinine clearance**			**Blood urea nitrogen**		
	**1st Week**	**2nd Week**	**3rd Week**	**1st Week**	**2nd Week**	**3rd Week**
**NORMAL**	1.148 ± 0.07			13.76 ± 1.4		
**CONTROL**	0.358 ± 0.09	0.429 ± 0.09	0.553 ± 0.07	41.14 ± 3.04	37.77 ± 1.45	36.81 ± 1.30
**N-MSC**	0.480 ± 0.04	0.584 ± 0.04	0.791 ± 0.04	33.79 ± 1.40	28.88 ± 1.16	24.95 ± 1.35
**SNAP**	0.581 ± 0.03*	0.796 ± 0.05*	1.008 ± 0.05*	28.10 ± 1.78*	21.80 ± 1.69*	14.58 ± 1.10*
**SNAP+MB**	0.432 ± 0.09	0.581 ± 0.08	0.792 ± 0.05	32.93 ± 0.61	28.13 ± 1.53	23.03 ± 0.92
**MB**	0.425 ± 0.08	0.578 ± 0.05	0.790 ± 0.05	34.16 ± 1.73	27.86 ± 1.03	23.14 ± 1.03

*= *p* is equal or less than 0.05.

N-MSC= Normal MSCs; SNAP= SNAP pre-conditioned MSCs; SNAP + MB=SNAP + methylene blue treated MSCs and.

MB= Methylene blue treated MSCs.

The values are mean ± SEM and analyzed by one way ANOVA.

**Figure 4:**
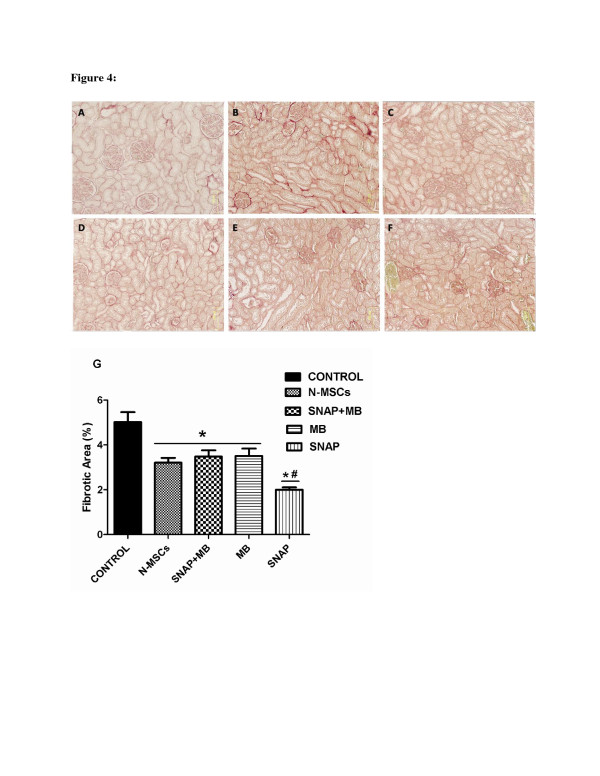
**Assessment of Fibrosis in ischemic kidney.A**-**F**: Representative micrograph of kidney tissue stained with Sirius red showing collagen deposition in various treatment groups (**A**-**F**) and (**A**= normal kidney, **B**= ischemic kidney, **C**= Normal MSCs, **D**= SNAP, **E**= SNAP + MB, **F**= MB and **G**= graphical presentation of quantitative analysis of fibrosis). #: *p < 0.05* SNAP *vs* MSCs, SNAP/MB and MB.

Fibrosis in ischemic kidney tissue was analyzed by sirius red staining which stains the fibrillar collagen content in a tissue. Three slides per animal and six fields per slide were analyzed for fibrosis measurement. Figure 4 (A-F) exhibits decreased fibrillary collagen in SNAP group compared to the other treatment groups. Quantitative analysis performed by Image J software (Figure 4G) indicates that pre-conditioning reduced the fibrosis in SNAP group while no significant reduction in fibrotic area was seen in other groups when compared to the ischemic control.

#### Effect of Pre-conditioning on proliferation of tubular cells

**Figure 5:**
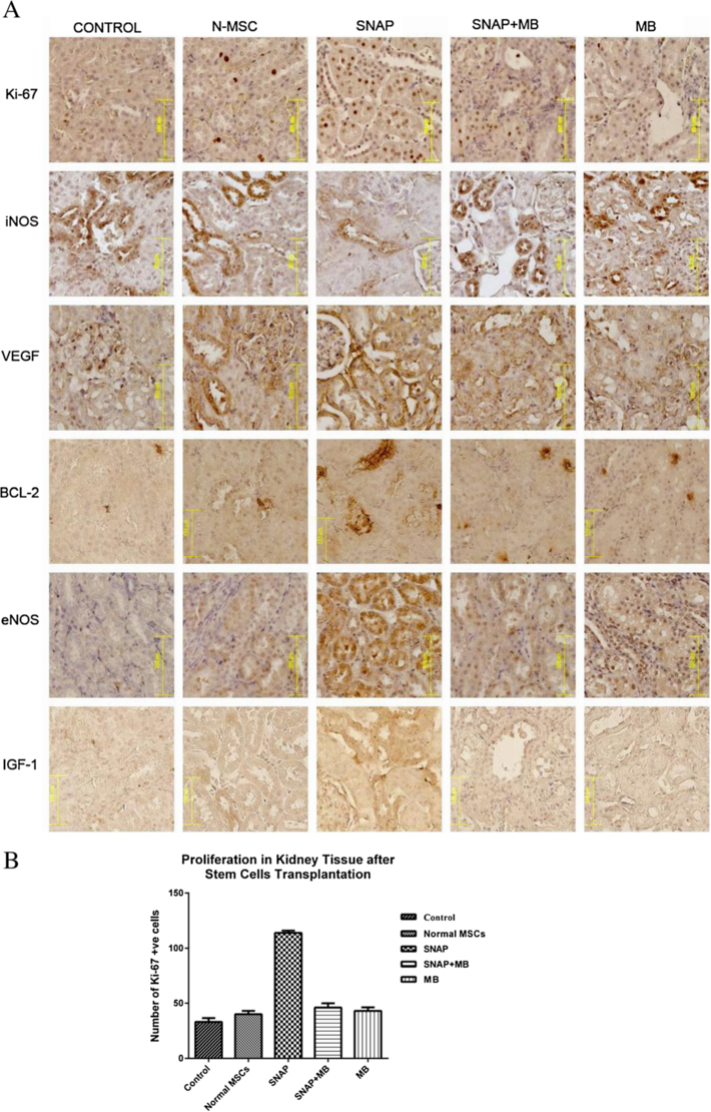
**Histology of renal tissue after MSCs transplantation.A**: Representative micrographs of renal histology after pre-conditioned MSCs transplantation. The expression of different pro-survival, pro-angiogenic factors, and proliferation was markedly increased in SNAP group compared to other treatment groups. Magnification 200x. **B**: Graphical presentation of Ki- 67 positive nuclei in different treatment groups.

The transplantation of SNAP pre-conditioned MSCs significantly enhanced the proliferation of tubular cells as depicted by Ki 67 expression in kidney. It was revealed that SNAP group had numerous Ki 67 positive nuclei compared to control group (114.0 ± 2.05 vs. 33.0 ± 2.01) while normal MSCs group, SNAP/MB and MB groups (Figure 5A and B) did not show such proliferation in kidney tissue (40.0 ± 2.36, 46.0 ± 2.7 and 43.0 ± 2.1).

#### Pro-survival and Pro-angiogenic factors induced by Pre-conditioned MSCs in ischemic kidney tissue

Among three isoforms of NOS inducible Nitric Oxide Synthase (iNOS) is induced in response to different patho-physiolocial conditions and acts independent to the release of calcium. iNOS aggravates the ischemia reperfusion injury in the kidney. Transplantation of SNAP pre-conditioned MSCs in the ischemic kidney tissue resulted in decreased iNOS levels compared to the ischemic control and treatment control (Figure 5). This decrease in the expression of iNOS was not seen in SNAP/MB and MB groups.

Bcl-2, IGF-1 and VEGF are cyto-protective growth factors and impart a vital role in cell survival, angiogenesis and vasculogenesis in the tissue. Expression of these factors in transplanted groups was analyzed by immunohistochemical staining. SNAP pre-conditioned group showed enhanced Bcl-2, IGF-1 and VEGF levels in ischemic kidney tissue compared with ischemic control, SNAP/MB and MB treated MSC transplanted groups. Concurrently the expression of eNOS, a constitutive form of nitric oxide synthase, expressed in endothelial cells was also found to be increased in the ischemic kidney tissue after SNAP group (Figure 5).

## Discussion

Bone marrow derived MSCs have the ability to ameliorate renal ischemia [19]. During renal ischemia, kidney becomes rich in reactive oxygen species, pro inflammatory cytokines and factors facilitating apoptosis [20]. Transplanted MSCs encounter such harsh environment resulting in poor survival which limits their utility in regenerative cellular therapy for renal ischemia [21]. In an attempt to improve the survival after transplantation, MSCs were pre-conditioned with a NO donor SNAP. NO is diverse biomolecule with a range of physiological functions and importance. NO has been found cyto-protective *in vivo* as well as *in vitro* against a variety of cyto-toxic agents [8,22-24]. Pre-conditioning of MSCs with 100 μM SNAP has multiple effects. It significantly reduced the cyto-pathic effects induced by H_2_O_2_ by improving the cell viability and survival *in vitro* (Figure 1). Pre-conditioning with SNAP improved the survival and engraftment of MSCs in ischemic renal tissue (Figure 3). Pre-conditioning may stimulate endogenous gene expression which enhanced their engraftment. It has been reported that NO regulates hemodynamics during renal organogenesis [25]. During ischemia reperfusion injury, iNOS is up-regulated and aggravates the renal injury which may be due to the production of peroxynitrites [26]. Our *in vivo* data showed that iNOS expression in kidney was reduced after pre-conditioned MSCs transplantation. Consequently fibrosis in ischemic renal tissue was reduced due to decreased collagen deposition in pre-conditioned MSCs transplanted group (Figure 4).

Akt activates many signaling cascades leading to regulation of a range of critical cellular functions like glucose metabolism, cell proliferation and survival [27]. NO mediates cyto-protection of isolated islets in serum deprivation by triggering PI3K/Akt pathway [28]. The protection mechanism of SNAP pre-conditioned MSCs seems due to activation of Akt pathway in these cells. Akt was up-regulated in SNAP pre-conditioned MSCs resulting in improved cell viability and reduced apoptosis *in vitro*. It enhanced expression of Bcl-2 in kidney of SNAP pre-conditioned group of animals, inhibiting apoptosis and facilitates cellular survival in the ischemic atmosphere of kidney by maintaining mitochondrial membrane integrity as shown previously [29]. Growth factors and cytokines play a pivotal role in development, cell differentiation, survival and proliferation. SNAP pre-conditioning induced up-regulation of an array of cyto-protective and pro-angiogenic cytokine and growth factor genes (IGF-1, SDF-1 and VEGF) in MSCs (Figure 2). IGF-1, SDF-1 and VEGF are cytoprotective, renotropic and pro-angiogenic growth factors playing an important part in vasculogenesis, angiogenesis and chemoattraction [19,30-33]. Up-regulated expression of VEGF in many cell types like smooth muscle cells, endothelial cells and keratinocytes is under the influence of NO [34-37]. VEGF along with Akt enhances the production of NO by activating eNOS leading to relaxation in endothelium and increased endothelial permeability which causes vasodilation of blood vessels [38]. Our results are in agreement with the previous studies as *in vitro* and *in vivo* data showed increased VEGF and eNOS expression in SNAP pre-conditioned MSCs but this response was abolished by pre-treating MSCs with methylene blue. Enhanced expression of VEGF and eNOS may result in the tissue protection by improved angiogenesis and perfusion leading to enhanced nutrient supply. Further, VEGF and SDF-1 could guide transendothelial migration of MSCs [39,40] into the ischemic renal tissue and their proliferation, but methylene blue treatment inhibited these factors. This whole milieu of up-regulated growth factors, chemokine, Bcl-2 and Akt may favor the maintenance of mitochondrial integrity and cell survival ensuing to renal protection against ischemia.

Proliferation is one of the important aspects in cellular therapies in order to repopulate the damaged tissue. In our studies, PCNA expression was higher which shows that SNAP Pre-conditioning elicited proliferation in MSCs *in vitro* (Figure 2) [41]. Concurrently, there was marked increase in Ki-67 expression in tissue sections from pre-conditioned MSCs transplanted kidneys (Figure 5A and B) compared to all other treatment groups showing early make up of cellular loss in kidney parenchyma resulting in improvement of renal function. This increase in proliferation activity was diminished by methylene blue treatment *in vitro* and *in vivo.*

Tubular epithelium is lost dramatically in ischemia reperfusion injury, resulting in impaired kidney function [42]. Our results demonstrated that mutilation of kidney function caused by ischemia was overcome by the transplantation of SNAP pre-conditioned MSCs in a better way by increased creatinine clearance and reduced BUN (Table 2) which could be due to improved perfusion and tubulogenesis.

## Conclusion

This study demonstrates that pharmacological pre-conditioning of MSCs plays a major part in augmenting resistance of MSCs to the oxidative stress *in vitro* and *in vivo* injury. Up-regulation of many cytoprotective factors help MSCs survive better in renal ischemic environment by activating Akt cell signaling. MSCs’ homing was improved in ischemic renal tissue leading to enhanced perfusion and tubulogenesis and improved renal function. Hence, pre-conditioning can be a potential strategy to enhance the outcomes of cell based therapies in attenuating renal ischemia injury.

## Competing interests

The authors declare that they have no competing interests.

## Authors’ contributions

MSM participated in the design of study, perform culturing and *in vitro* treatments, participated in *in vivo* experiments, executed statistical analysis and drafted the paper. SSA carried out polymerase chain reaction. MZA performed *in vitro* immunoassays and helped to draft the manuscript. AM analyzed the *in vivo* data and helped to draft the manuscript. SNK participated in study design and proofreading the manuscript and SR contributed in study design, funding and final approval of the manuscript. All authors read and approved the final manuscript.
